# A Convention of Confederate Surgeons

**Published:** 1874-04

**Authors:** 


A Convention of Confederate Surgeons is called, by S. P. Moore, M.D., late Surg. Gen., P. A. C. S., to meet in Atlanta, Ga., May 20th, prox. We trust the Confederate Surgeons throughout the South, will meet in response to the call, as the object is to gather and preserve the Medical and Surgical History of the Confederate Army. W. T. G.

Dr. B. T. G.:—I have not forgotten my promise to write a short article on Policy and Slander. I will try soon. The third subject suggested by our friend, Dr. H., I must decline giving any opinion. It would be useless. What is in the bone can not be got out of the flesh. To attempt to learn some men something, would be like "casting pearls before swine." P.

